# Knee arthrodesis using a unilateral external fixator combined with crossed cannulated screws for the treatment of end-stage tuberculosis of the knee

**DOI:** 10.1186/s12891-015-0667-2

**Published:** 2015-08-19

**Authors:** Xin Tang, Jing Zhu, Qi Li, Gang Chen, Weili Fu, Jian Li

**Affiliations:** Department of Orthopedic Surgery, West China Hospital of Sichuan University, No.37, Guoxue Alley, Chengdu, 610041 P.R. China; Department of Respiratory and Tuberculosis Care Medicine, West China Hospital of Sichuan University, No.37, Guoxue Alley, Chengdu, 610041 P.R. China

## Abstract

**Background:**

The treatment of end-stage tuberculosis (TB) of the knee remains a significant clinical challenge, and clinical data are lacking. This study aimed to retrospectively determine the outcome of single-stage knee arthrodesis with a unilateral external fixator combined with cannulated screws for the treatment of end-stage TB.

**Methods:**

Twenty-six patients with end-stage knee TB were treated by single-stage arthrodesis. All patients underwent open debridement and the insertion of a unilateral external fixator combined with crossed cannulated screws and received systemic antitubercular therapy. Clinical evaluations and radiographic analyses were performed after an average follow-up duration of 5.5 years.

**Results:**

The mean time to radiographic bone fusion was 5.6 months. Primary full union was achieved in 25 patients (96.2 %) within 8 months, and the remaining patients achieved bone fusion at 14 months postoperatively. The mean post-operative alignment was 5.4° valgus and 12.5° flexion. The mean leg-length discrepancy was 2.8 cm. The mean VAS score improved from 67.3 before surgery to 16.2 at the last follow-up (*P* < 0.01), and the mean WOMAC score improved from 58.8 to 13.7 (*P* < 0.01). The erythrocyte sedimentation rate (ESR) and C-reactive protein level returned to normal in 25 patients by 3 months postoperatively. No recurrence in the target knee was noted.

**Conclusions:**

Single-stage arthrodesis with a unilateral external fixator combined with cannulated screws can be regarded as efficacious for the treatment of end-stage knee TB. Additional studies are necessary to confirm the findings of our study.

## Background

The prevalence of tuberculosis (TB) remains high in China [1]. In 2010, an estimated one million new tuberculosis cases were reported in this country, accounting for 11 % of the global TB incidence [2]. Simultaneously, cases of extrapulmonary osteoarticular TB have shown an increasing trend [3, 4], with the spine being the most common skeletal site (50 %), followed by the hip and knee joints (10 % each) [5]. Treatment of TB arthritis with appropriate and timely chemotherapy can result in a good outcome [6]. However, it is difficult to accurately diagnosis knee TB arthritis in adults due to its rarity and lack of characteristic symptoms, signs and imaging manifestations in early stage patients [7, 8]. The delayed treatment of knee TB arthritis may result in total knee TB with severe cartilage destruction or high-grade joint deformation [9].

Treatment of end-stage knee TB remains a significant clinical challenge. Currently, open debridement and arthrodesis are the main treatment options for this condition in adults [6, 10]. Literature reviews have reported that a stable, painless knee can be obtained in a patient after a failed arthroplasty, septic arthritis, or recurrent infection [11, 12]. Various techniques have been described, including external fixation, internal fixation with an anterior plate or double plating, an antegrade locking nail, modular intramedullary nails, etc. [11–16]. However, no single technique has been proven to be superior for treating TB-infected patients. Moreover, to our knowledge, there are few reports of the use of these techniques for the treatment of end-stage knee TB.

Patients with end-stage knee TB have a low rate of fusion with a high incidence of complications. We attempted to identify a simple and effective solution for these patients. In this retrospective study, we aimed to evaluate the efficacy of single-stage knee arthrodesis with a unilateral external fixator combined with cannulated screws for the treatment of end-stage knee TB.

## Methods

This study was approved by the Human and Ethics Committee for Medical Research at Sichuan University in accordance with the Declaration of Helsinki. Written informed consent was obtained for all patients prior to their inclusion in the study.

Twenty-six patients with end-stage knee TB who underwent single-stage arthrodesis of the knee using a unilateral external fixator combined with cannulated screws (Weigao, China) between 2005 and 2011 were evaluated retrospectively. The main characteristics of the 26 patients are described in Table 1. There were 12 men and 14 women, with a mean age of 43.7 years (range, 21 to 72 years) at the time of arthrodesis. The mean duration of illness was 3.5 years (range, 1 to 7.5 years).

**Table 1:** TB patient characteristics

Characteristics	TB, *n* = 26
Age,years, mean ± SD	43.7 ± 11.7
Sex, M/F	12/14
Side,L/R	15/11
Duration, years, mean ± SD	3.5 ± 1.9
Pre-operation ESR, mm/h, mean ± SD	46.2 ± 32.2
Pre-operation CRP, mg/L, mean ± SD	37.0 ± 35.9
Pre-operation VAS score	67.3 ± 11.2
Pre-operation WOMAC score	58.8 ± 8.1
Follow-up time, years, mean ± SD	5.5 ± 1.9

*TB* tuberculosis; *ESR* erythrocyte sedimentation rate; *CRP* C reactive protein; *VAS* visual analogue scale; *WOMAC* Western Ontario and McMaster Universities’ Osteoarthritis Index

Of these patients, 15 also had a TB abscess around the knee, while 7 had complications with sinus and mixed bacterial infections, including 1 concurrent sinus infection around the knee and lateral trochanter. Gram-positive cocci were predominant in five of the seven patients with mixed bacterial infections (3 *Staphylococcus aureus*, 1 *Staphylococcus epidermidis*, and 1 *Staphylococcus aureus* and *Enterococcus*), and gram-negative bacilli were predominant in two patients (2 *Escherichia coli*).

Of these patients, 11 had been diagnosed with knee TB previously and had received arthroscopically assisted knee debridement at local hospitals. Two patients were experiencing recurrences who had undergone previous fusion with simple crossed cannulated screw fixation after arthroscopically assisted knee debridement at our hospital; three patients had been diagnosed with knee TB and had received open knee debridement but no fusion at local hospitals; six patients had been diagnosed with end-stage knee TB at our department but had not received any previous operation; and the remaining four had been misdiagnosed preoperatively as having supportive arthritis (1 case), gouty arthritis(1 case) and pigmented villonodular synovitis (PVNS) (2 cases) but were then shown to have TB by operative synovial fluid polymerase chain reaction testing or pathologic analysis of biopsy specimens (or both).

**Fig. 1:**
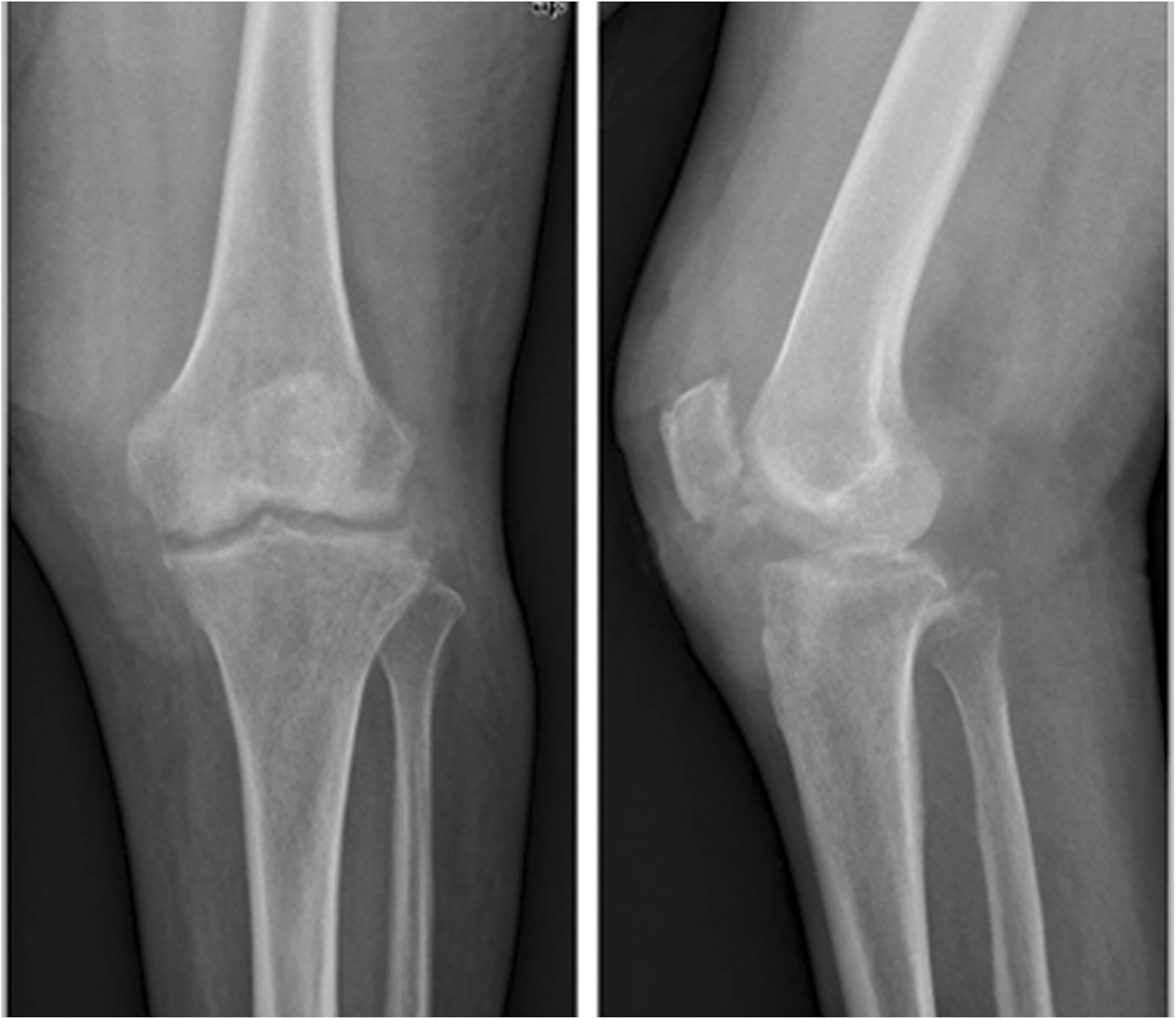
X-ray (anteroposterior and lateral) showing clearly osteoporosis and joint destruction

**Fig. 2:**
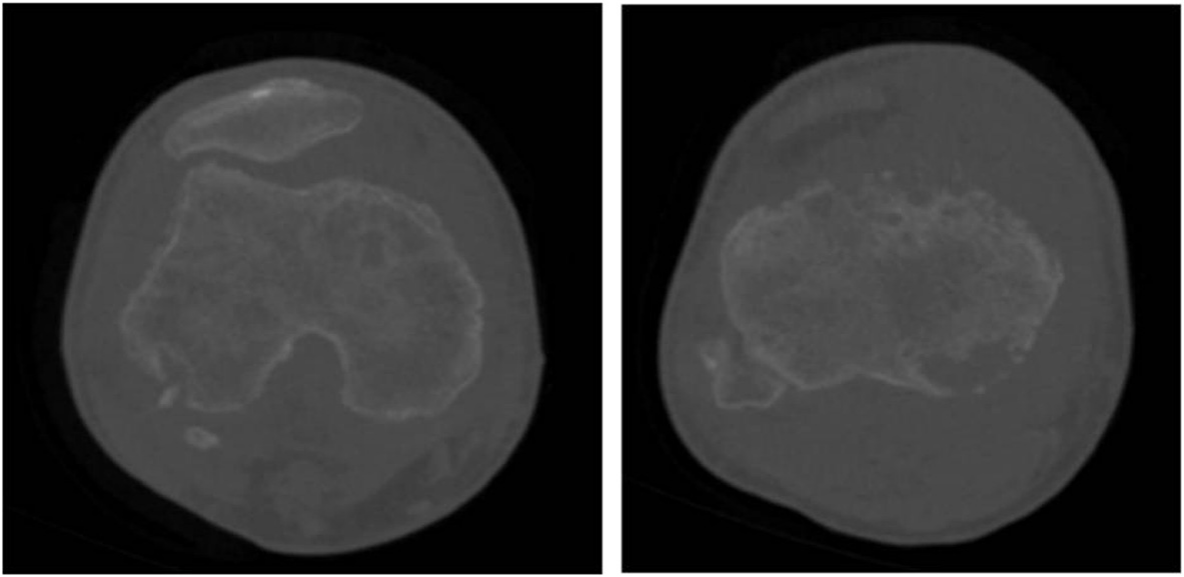
CT (axial) showing severe joint destruction and central and peripheral erosions

**Fig. 3:**
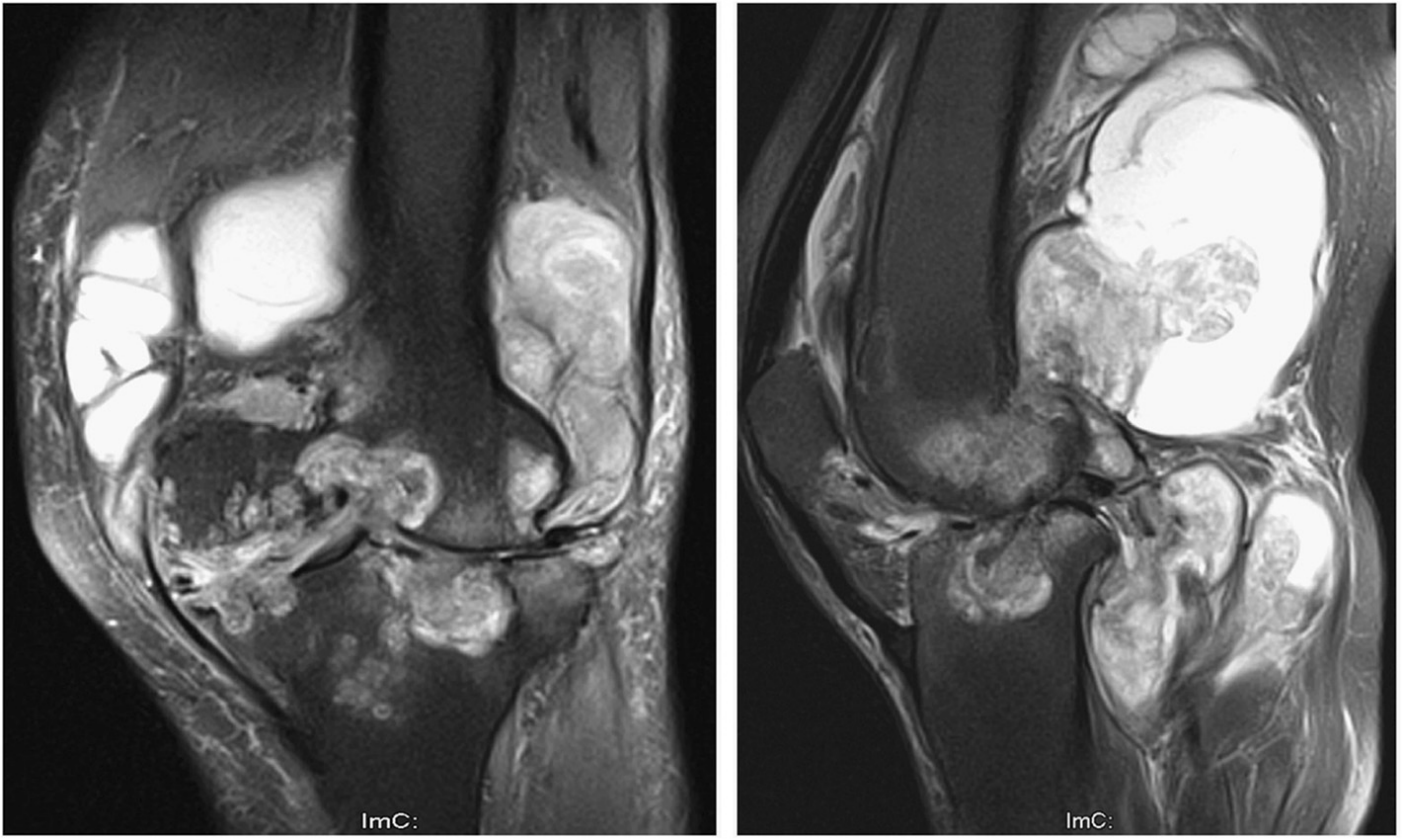
MRI (anteroposterior and lateral) showing severe joint destruction, obscure subcartilaginous bone plates, central and peripheral erosions, abscesses and articular cartilage damage

Before operation, the patients with TB infection typically shared some classical characteristics, including low-grade fever and night sweats, knee swelling and pain, previously diagnosed or currently active pulmonary TB, a high erythrocyte sedimentation rate (ESR), an increased level of C-reactive protein, a positive result for DNA sequencing of TB bacillus or a staining test for identifying acid-fast bacilli, a positive result on the purified protein derivative test, and a positive result on the blood TB antibody test. On the basis of radiograph, computed tomography (CT) and magnetic resonance imaging findings (Fig. 1, 2 and 3), all of the patients shared some classical characteristics, including severe joint destruction, eventually leading to fibrous ankylosis, central and peripheral erosions, various degrees of osteoporosis, obscure subcartilaginous bone plates or small amounts of articular hydrops, abscesses, bone chips, and articular cartilage damage [17]. Patients with more than two above characteristics combined with essential clinical manifestations before operation were suspected to have TB. Some patients underwent diagnostic treatment with anti-TB drug.

The final diagnosis was confirmed by postoperative pathology for all patients. The histopathologic results mainly showed synovial papillary proliferation, tuberculosis nodules (cheese necrosis, Langhans giant cells, epithelial cells and inflammatory cells) and granulomatous lesions (epithelioid histiocytes surrounded by lymphocytes, granulomas fibrosis and calcification).

### Surgical technique

Before surgery, all patients preoperatively diagnosed with TB (including those with suspected TB) received anti-TB drug therapy (rifampin (450 mg/d) + isoniazid (300 mg/d) + ethambutol (750 mg/d)) for at least 3 weeks until the ESR became stable or declined. Intramuscular treptomycin was administered for at least 3 weeks to the patients without an allergic reaction. For those patients who experienced a rise in the ESR within 3 weeks after the initiation of the anti-TB drug therapy, we increased the duration of therapy before operation until the ESR became relatively stable or declined. Alternatively, we considered these patients to have complicated drug-resistant TB and adjusted the treatment program based on advice from TB experts at our hospital, such as the addition of oral levofloxacin or replacement rifampicin with rifapentine. The patients with mixed bacterial infections received sensitive antibiotic treatments until the infection was under control. The patients with anemia and hypoalbuminemia were preoperatively encouraged to eat and to receive intravenous supplementation or other methods of supplementation before surgery until these conditions returned to normal.

**Fig. 4:**
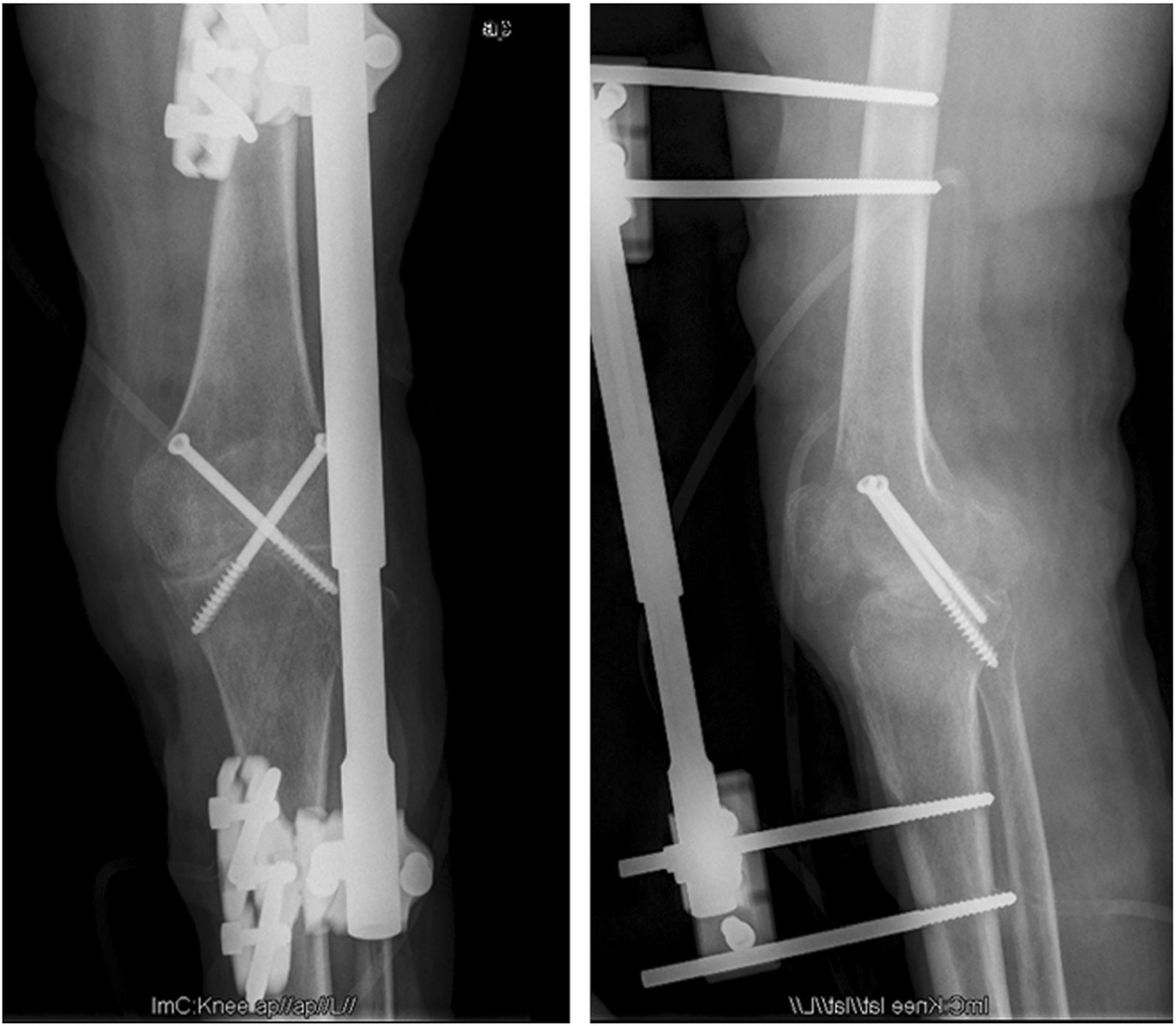
Good bony contact shown on radiographs (anteroposterior and lateral) after operation

The patients were placed in the supine position and administered lumber plexus and sciatic nerve block anesthesia. A tourniquet was used on the upper leg (pressure of 280 mm Hg). Either a midline incision was created or a previous incision was used, as dictated by the condition of the skin under the tourniquet. Skin flaps were not disturbed more than necessary to minimize damage to cutaneous circulation. The patella was preserved if possible in preparation for two-stage joint replacement surgery. The posterior region of the knee was exposed by pulling the femur up and pressing the tibia back or pulling it forward. The menisci, cruciate ligaments and any debris were thoroughly excised. Samples of fibrous tissue and bone were collected for microbiological studies. If present, sinuses were excised thoroughly. Any abscess near the primary incision was cleared through this incision, while any abscess far from this incision was cleared by creating an additional incision. After thorough debridement, the wound was flushed and then soaked in povidone-iodine for a quarter of an hour before washing. The bone ends were prepared by removing all articular cartilage and preparing the appropriate surfaces for apposition. To maintain limb length, minimal bone resection was performed. The femur and tibia were placed in 5° to 7° valgus and 10° to 15° flexion and were temporarily fixed with two crossed Kirschner wires positioned from the medial or lateral condylus of the femur to the lateralor medial condylus of the proximal tibia. Once the optimal position of fusion was confirmed under fluoroscopy, two crossed cannulated screws were inserted along with two temporary fixed Kirschner wires to obtain equal compression in the coronal plane. Then, the incision was closed after adequate hemostasis and lavage, a drain was placed, and the leg was dressed in extension prior to releasing the tourniquet. Next, a further unilateral external fixator was added in front of the knee for the resistance of shear stress in the sagittal plane (Fig. 4).

### Postoperative management and assessment

Immediately after surgery, all patients were continued on anti-TB drug therapy (rifampin (450 mg/d) + isoniazid (300 mg/d) + ethambutol (750 mg/d)) for 12 months or until the serum levels of inflammatory markers became normal (testing was continued at least three times per week at 2 week intervals). They also continued on intramuscular streptomycin treatment (75 mg/d) for 1 month after operation in the absence of an allergic reaction. The patients with mixed bacterial infections were continued on sensitive antibiotic treatment until the wound healed.

The patients were able to walk with partial weight-bearing at 2 days postoperatively and with full weight-bearing at 8 to 12 weeks postoperatively. The external fixator was removed when union was observed on radiographs.

All of the patients were assessed both clinically and radiologically. Pain was assessed using a visual analog scale (VAS) using scores ranging from 0 to 100, with 100indicatingthe highest level ofpain. The functional outcome was determined by the Western Ontario and McMaster Universities Osteoarthritis Index (WOMAC, version LK3.1) subscale, which is scored on a five-point Likert scale (none = 0; mild = 1; moderate = 2; severe = 3; and extreme = 4) [18]. Serial radiographs were used to determine the time of fusion. Clinical and radiographic union was defined as the ability to walk without pain or tenderness at the arthrodesis site and the appearance of a circumferential bridging callus on both the anteroposterior and lateral films [19]. In addition, the limb alignment and leg-length discrepancy were measured on full-length radiographs.

The C-reactive protein (CRP) level, ESR, and white bloodcell counts were determined at each visit, followed by a thorough clinical evaluation.

### Statistical analysis

Statistical analyses were conducted with SPSS Version 16.0 software (SPSS Inc., Chicago, IL, USA). The distributions of all measurement variables were determined with the Kolmogorov-Smirnov test before statistical analyses were performed. Comparisons of variables between baseline and the endpoint were analyzed using paired t-tests when the distribution was normal; otherwise, the Wilcoxon signed-rank test was used. A p value of less than 0.05 was considered significant and that of less than 0.01 was considered highly significant for all of the statistical tests.

## Results

The mean follow-up time was 5.5 years (range, 3 to 8 years). All of the patients were followed-up for at least 2 years after their operation. Four patients were lost to follow-up at 2 years after surgery because their knee replacement was successful and they did not want to return for follow-up.

**Fig. 5:**
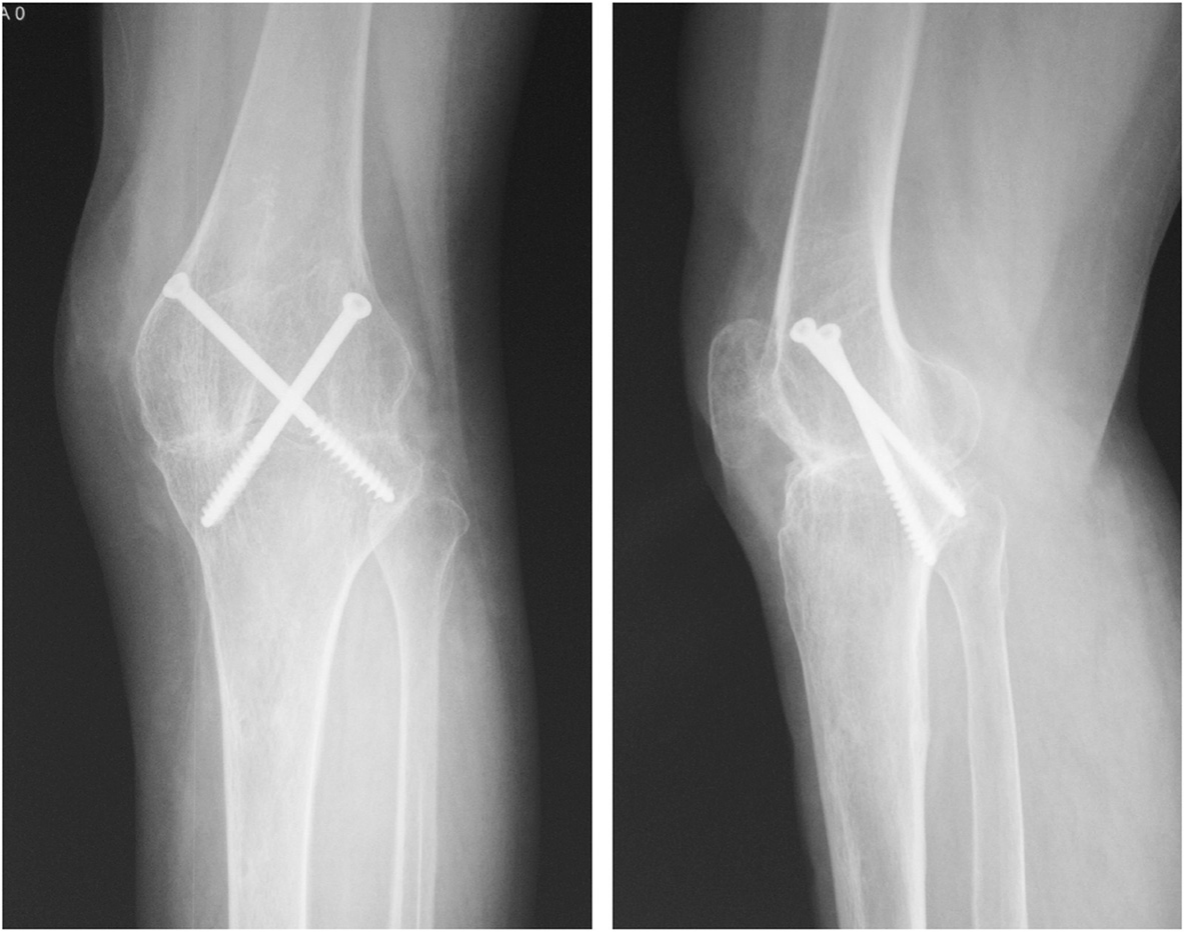
Good knee fusion shown on radiographs (anteroposterior and lateral) after removal of the external fixator

The mean time to radiographic bone fusion was 5.6 months (range, 4 to 14 months). Primary full union was achieved and the external fixator was removed in 25 patients (96.2 %) within 8 months after operation, as shown by the radiographs (Fig. 5). The remaining patient achieved only partial bone fusion in the interior tibio femoral joint at 8 months. Fortunately, this patient obtained full fusion at 14 months postoperatively after a prolonged external fixator time, and it was then removed.

**Fig. 6:**
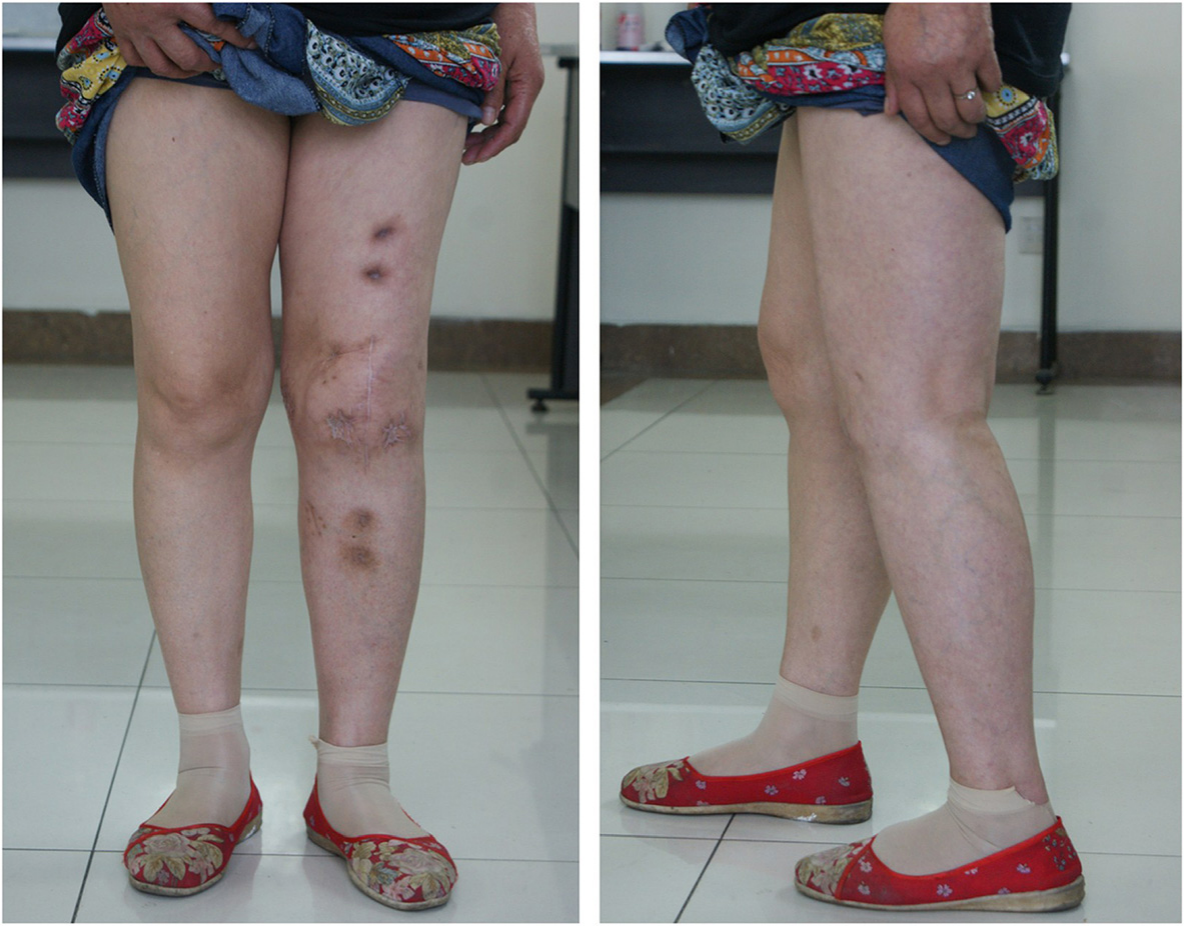
Good alignment shown atthe anteroposterior and lateral positions after removal of the external fixator

Long-leg film analysis (Syngo Imaging V31, Siemens AG Medical Solutions, Germany) revealed that the mean post-operative alignment was 5.4° valgus (range, 0.9 to 8.5°) and 12.5° flexion (range, 7.3 to16.9°) (Fig. 6). The mean leg-length discrepancy was 2.8 cm (range, 1.0 to 5.5 cm) after fusion. No patient had a significant rotational deformity.

The mean VAS score improved from 67.3 (range, 55.0–85.0) for walking before surgery to 16.2 (range, 0–30.0) at the final follow-up (*P* < 0.01, as determined by the paired *t*-test). The mean WOMAC score improved from 58.8 points (range, 45.0 to 78.0 points) before surgery to 13.7 points (range, 9.0to 25.0 points) at the final follow-up (*P* < 0.01, as determined by the paired *t*-test).

The ESR and CRP level returned to normal in 25 patients by 3 months postoperatively. One patient developed a new sinus on the lateral greater trochanter of the femur at 3 months after surgery and had to receive further debridement and an additional course of anti-TB drug therapy. Wound healing was achieved one month later, and no recurrence was noted. Three patients suffered from pin tract infections after surgery on the femoral side. These infections were relieved following administration of oral antibiotics and wound care, and they did not affect the bone union of the knee.

## Discussion

Arthrodesis is the traditional treatment for end-stage TB of the knee. The techniques that are available to achieve arthrodesis of the knee include intramedullary fixation, external fixation, and plating [11, 12]. However, no single technique has been proven to be superior for the treatment of end-stage knee TB, and no description of single-stage knee arthrodesis for the treatment of end-stage TB arthritis using a combined technique has been reported.

In our study, the rate of primary bone union at the final follow-up was 96.2 %, and one patient had delayed union but did not require a secondary procedure. The mean time to union was 5.6 months. These data are in agreement with those reported by previous studies, which have used various fixation techniques for treating infected knees after arthroplasty to achieve arthrodesis, reporting fusion rates and times ranging from 68 to 100 % and 4.5 to 9.9 months, respectively [13–16, 20–26]. Notably, we obtained good clinical outcomes in the current study. The mean WAS and WOMAC scores observed at the final follow-up improved significantly compared with those determined before knee arthrodesis was performed, similar to the results of Bargiotas K [13]. The ESR and CRP level were restored to normal in 96.2 % of the patients at 3 months postoperatively, which is consistent with Tang’s report on the ESR and CRP level following ankle fusion in patients with end-stage TB [17]. No recurrences occurred during the follow-up period in the current study, except for one patient with an extra-articular sinus complication.

Each technique has advantages and disadvantages. Intramedullary nailing and plating can result in a high rate of union and excellent stability, but the necessary surgical techniques are challenging and require extensive exposure of the joint, which increases the risk of the dissemination of infection [12, 14, 27, 28]. In fact, internal fixation techniques should not be considered for any knee infection, including knee TB. External fixation was popularized as a method of arthrodesis and was indicated for the treatment of TB arthritis. In this method, the pins do not pass through the lesions, minimizing the possibility of TB dissemination. A single-plane external fixator, such as the Charnley device, is convenient for the treatment of some knee infections but provides minimum stability and is associated with a lower healing rate [29]. Circular external fixators, such as the Ilizarov device, provide the greatest stability and allow for immediate weight bearing in patients, but the full ring at the distal part of the femur needs to be very large to accommodate the abundant soft tissues. The large volumes, pin loosening and pin tract infections associated with these fixators may inconvenience patients, resulting in their dissatisfaction [11, 22].

Arthrodesis using simple cannulated screws is technically straight forward, convenient for patients, and is associated with low rates of delayed union and infection recurrence, and compression can be obtained [30]; however, this technique does not provide sufficient fixation and usually requires immobilization during the early postoperative stages, and full weight bearing is delayed until fusion has occurred. The main reason for immobilization after surgery is because simple compression fixation using cannulated screws in the coronal plane does not provide sufficient stability or shear stress resistance in the sagittal plane. Therefore, we added a further unilateral external fixator at the front of the knee to achieve this resistance. The technique we report here is intended to increase rigidity while limiting the size of the external fixator and the number of pin channels, thereby minimizing the risk of TB dissemination and decreasing the risk of pin infection.

Knee arthrodesis with a unilateral external fixator combined with crossed cannulated screws for the treatment of end-stage TB has some advantages, as follows: (1) it provides various modes of mechanical action, compression fixation and stable fixation, which allow for full weight bearing during the early postoperative stages; (2) external fixation occurs away from the affected site, minimizing the possibility of TB dissemination and allowing for better wound management; (3) open cannulated screw fixation facilitates other processes of the operation, and the external fixator is inserted after releasing the tourniquet and closing the incision, which reduces the durations of surgical and tourniquet exposure; and (4) even when the external fixator is removed in the case of pin tract infection or pin loosening, the ideal position can still be retained due to the internal fixation, and an additional reduction procedure is typically not required.

Complete debridement is crucial for the treatment of TB arthritis, which can eradicate infected tissues to lower the risk of TB reactivation. However, debridement is usually performed as a basic procedure for treating knee TB and is typically more effective for early stage knee TB [31–33]. Patients with end-stage TB arthritis with severe joint damage and periarticular abscess who undergo a single debridement may have a relatively higher recurrence rate because the control and eradication of the infection is very difficult using this single surgical approach combined with preoperative and post-operative anti-TB drug therapy [6, 34]. These findings have been confirmed in our study, in which a 61.5 % (16/26) recurrence rate was observed after debridement (11 cases of arthroscopic debridement and 3 cases of open debridement).

Knee arthroplasty can result in a good or excellent functional outcome for the treatment of end-stage knee TB [34–38]. Several novel studies have also been conducted evaluating the treatment of patients with active TB arthritis of the knee with total knee arthroplasty (TKA)[34, 35, 37]. However, the risk of reactivation of TB arthritis should cause orthopedic surgeons to be reluctant to perform this procedure immediately after debridement [39–41]. There are always some patients who experience persistence or reactivation of TB infection after arthroplasty [39–41]. Importantly, our study was performed at a remedy center in southwest China, where most of the TB patients were from remote and poor areas. Financial constraint is the key reason for patients with knee TB to delay treatment. As a result, many patients progress to end-stage TB with severe articular damage and periarticular abscess with or without sinus tracts. Therefore, we must consider a simple and effective technique that not only cures TB infection but also is inexpensive. The unilateral external fixator combined with crossed cannulated screws meets both of the above mentioned criteria. In addition, the current worsening of the doctor-patient relationship in China may prevent orthopedic surgeons from providing single-stage TKA for the treatment of end-stage TB arthritis due to the risk of possible reactivation of TB infection.

Arthrodesis may be more appropriate for patients with minimal bone loss and broad cancellous surfaces, which permit good bony apposition and compression [30]. However, for those with destructive and massive bone defects that arise after radical debridement, the serious shortening of the limb may cause discomfort and also restrict activities of daily living. Thus, a second-stage TKA may be the safest option. We cannot conclude that a unilateral external fixator combined with crossed cannulated screws is the best method for treating end-stage knee TB, although it may be one of the best methods for patients from less-developed regions. Certainly, some patients can receive a successful second-stage total knee replacement when their economic condition improves, which has occurred for several patients in our study. For some failed TKAs in patients with uncontrollable tuberculous arthritis, knee arthrodesis can be viewed as the last option for treatment as a salvage method and may serve to avoid amputation, which has been confirmed in patients with failed TKA as the result of infection [11, 13, 14, 21, 42]. Literature reviews also have reported that a stable, painless knee can be obtained in a patient after a septic arthritis, or recurrent infection [11, 12].

A limitation of our study is that we used a small sample size and a short follow-up period. In addition, this study was retrospective. Therefore, we could not draw any firm conclusions. A large, prospective, randomized, controlled study is needed. However, to our knowledge, this study includes the largest sample size among all retrospective and prospective studies evaluating knee fusion for the treatment of end-stage knee TB.

## Conclusions

Based on our study, single-stage arthrodesis of the knee using a unilateral external fixator combined with crossed cannulated screws can be regarded as efficacious for the treatment of end-stage knee TB, and it is associated with a high rate of union, minimal complications and patient convenience. Further extensive studies are needed to confirm the findings of our study.

## Abbreviations

TB: Tuberculosis
VAS: Visual analog scale
WOMAC: The Western Ontario and McMaster Universities Osteoarthritis Index
ESR: Erythrocyte sedimentation rate
CRP: C-reactive protein
